# Heart Rate Variability’s Association with Positive and Negative Affect in Daily Life: An Experience Sampling Study with Continuous Daytime Electrocardiography over Seven Days

**DOI:** 10.3390/s23020966

**Published:** 2023-01-14

**Authors:** Justin Hachenberger, Yu-Mei Li, Michael Siniatchkin, Katharin Hermenau, Sebastian Ludyga, Sakari Lemola

**Affiliations:** 1 Department of Psychology, Bielefeld University, 33615 Bielefeld, Germany; 2 University Clinic for Child and Adolescent Psychiatry and Psychotherapy, Protestant Hospital Bethel, University Clinics OWL, 33617 Bielefeld, Germany; 3 Department of Sport, Exercise and Health, University of Basel, 4001 Basel, Switzerland; 4 Department of Psychology, University of Warwick, Coventry CV4 7AL, UK

**Keywords:** psychophysiology, momentary assessment, ambulatory assessment, intensive longitudinal data, electrocardiogram, affect, mood, heart rate variability

## Abstract

Heart rate variability has been found to be related to emotional processing and emotional responses. Studies that investigated these relationships were mostly lab-based or cross-sectional. Only limited research used intensive longitudinal data, in particular investigating within-individual processes in real-life settings. This study addresses the applicability of ambulatory-assessed electrocardiograms in combination with the experience sampling methodology by investigating the associations of various HRV measures with affective states on within- and between-individual levels. A total of 26 participants aged 18–29 years (23 females) wore electrocardiograms continuously for seven days. The participants received seven prompts per day and answered questions about their affective wellbeing. The heart rate and heart rate variability measures differed between body positions and activity classes. The heart rate and ratio of low-to-high-frequency heart rate variability were consistently associated with positive affect on a within-individual (state-like) level. These associations were mainly driven by the items of feeling “enthusiastic” and “happy”. No associations were found with negative affect. Overall, we found evidence that the dominance of the sympathetic nervous system over the parasympathetic nervous system was associated with higher levels of positive affect on a within-individual (state-like) level. Suggestions for the application of ambulatory electrocardiogram assessment in the study of the association between autonomous nervous system activity and ecological momentary assessment-based variables are discussed.

## 1. Introduction

The interconnection between emotional and somatic states has been widely studied. Among these is the autonomic nervous system (ANS), which is considered to play a major role in emotional responses [1]. The ANS is commonly divided into the parasympathetic (PNS) and sympathetic nervous systems (SNS), where the PNS is generally associated with resting and relaxation (e.g., decreasing heart rate; HR) while the SNS is associated with “fight-or-flight” responses and generally higher arousal and attention (e.g., increasing HR) [2,3]. A non-invasive marker of the ANS or of parasympathetic or sympathetic activity is heart rate variability (HRV), which represents the changes in interval length between adjacent heartbeats that can be recorded via electrocardiography [4].

### 1.1. HRV Measures, and Their Relationship with the PNS and SNS

Commonly, HRV measures are based either on frequency-domain or on time-domain methods. Frequency-domain methods determine the distribution (power) of interbeat interval lengths in different frequency bands, while time-domain methods quantify the variability of interbeat intervals [4]. HRV measures derived from the frequency domain include the power of the low-frequency band (0.04–0.15 Hz; HRV-LF), the power of the high-frequency band (0.15–0.40 Hz; HRV-HF), and the ratio of low- to high-frequency power (LF/HF ratio). Within the time domain, the root-mean-square of successive differences (RMSSD) between heartbeats plays an important role [4,5].

To date, HRV-LF power has been largely assumed to reflect SNS activity. However, this has recently been challenged by another notion. Namely, the relative contribution of the PNS and SNS to HRV-LF power depends on the measurement context [4]. More precisely, HRV-LF is reasonably representative of sympathetic activation in longer measurement contexts, but in short measurement contexts of only a few minutes in a resting state, sympathetic activation has been suggested to be less well represented [5]. HRV-HF, in contrast, appears to be mainly produced by the PNS [4]. The LF/HF ratio has traditionally been used to examine the ratio of sympathetic activity to parasympathetic activity [4,5]. In this view, a higher ratio is considered to represent SNS dominance, while a lower ratio is considered to represent PNS dominance. The RMSSD is highly correlated with the HRV-HF and appears to be strongly influenced by the PNS [4,5]. Typically, the short-term assessment of HRV and the derived scores are measured in or aggregated to 5 min epochs [4,6]. This was also recommended by the Task Force of the European Society of Cardiology and the North American Society of Pacing Electrophysiology [7]. Furthermore, the context of measurement, particularly the body position and the current activity status, must be considered. For example, changes from lying to upright or from physically inactive to physically active typically correspond to remarkable changes in SNS and PNS activity. To make the measurements comparable, it has been recommended that the measurements are taken in a resting state, i.e., when people are inactive and sitting [7,8].

### 1.2. Research Paradigms Used in Previous Research on the Relationship between Emotional States and the ANS

As measures for the ANS, HR and HRV have been studied extensively, particularly their associations with specific emotions [1]. The typical studies were lab-based and used paradigms in which emotional states were elicited by stimuli that were specifically chosen to evoke a certain type of emotional response. For instance, participants were instructed to watch pictures or film clips or to recall specific events in their lives that were associated with certain emotions. As an example of this type of study, Shi et al. [9] investigated the differences in a number of HRV parameters between happy and sad emotional states, which were elicited by two 7 min movie clips. They found that happiness was associated with higher HRV-LF, lower HRV-HF, and a higher LF/HF ratio compared with sadness. In this paradigm, within-individual differences in the HRV between experimental conditions are modelled statistically (e.g., “baseline” vs. “emotion stimulating condition”; or “happy condition” vs. “sad condition”).

A second type of research paradigm involves the individual differences paradigm, in which HRV measures and emotional states (or traits) are assessed by questionnaires in cross-sectional or longitudinal studies. An example is the study by Geisler et al. [10], who investigated participants’ mood and HRV-HF cross-sectionally, and found that cheerfulness and calmness were positively correlated with HRV-HF. While Geisler et al. [10] indicated that the HRV-HF measured over seven minutes in the resting state showed trait-like features with a re-test reliability of 0.70 over one hour, there were also considerable within-individual fluctuations, which might partly have been associated with within-subject fluctuations in mood states.

### 1.3. Studying the Association between Affective States and the ANS in Intensive Longitudinal Data

Intensive longitudinal data (ILD) are defined as data involving repeated measurements per participant including at least five measurement occasions [11]. When applying ILD in studying the associations between ANS activity and emotional states, it involves, for example, ambulatory assessment of HRV and measuring affective states with experience sampling methodology (ESM) over an extended period of time, ranging from a few hours to months. An advantage of the ILD approach over cross-sectional studies analysing between-subject variations is the possibility to disentangle the within-individual state-level variation and the between-individual trait-level variation. While researchers often tacitly assumed that between-individual variation reflects within-individual processes, evidence suggesting the contrary is accumulating [12,13]. Furthermore, an advantage of the ILD approach over lab-based studies, which involve triggering emotions with assorted stimuli, is that the ILD approach investigates affective states in natural settings and, therefore, has greater ecological validity. Thus, the ILD approach avoids potentially overemphasizing of one short lab session and investigates how psychological, physiological, and somatic processes unfold together over an extended period of time.

While there are many potential advantages of adopting the ILD approach in studying the interplay between emotional states and ANS activity, we are aware of only two studies that have used this approach. In a study by Schwerdtfeger and Gerteis [14], HRV was operationalised by RMSSD alone, which was found to be negatively associated with activated facets of positive affect, while it was positively associated with deactivated facets of positive affect. Another study by Kim et al. [15] focused on the associations of HR, RMSSD, and the standard deviation of RR intervals (intervals between successive heartbeats) with negative affect, and found significant associations with low-arousal negative affect (i.e., sluggish, bored, and sad), but not with high-arousal negative affect (i.e., anxious, annoyed, and upset).

The overall lack of ILD studies may be due to the challenges related to studying electrocardiograms (ECGs) in natural settings, particularly examining the associations between ECG recording segments with coinciding ESM measurements of psychological states. As described above, HRV measurements are partly determined by body position and physical activity; therefore, these variables have to be measured and controlled alongside the HRV and ESM measurements.

A second challenge relates to the assessment of emotions with ESM in ecological settings: A potentially important difference of affective states studied in the ESM compared with emotions elicited in lab-based studies is that in the ESM, rather general mood states or affective “tone” are measured, rather than the emotional responses to experimental stimuli. There have been attempts to trigger affect measurements specifically when emotional reactions occur in ESM research, e.g., by measuring changes in electrodermal conductivity (see e.g., [16]). However, there is also reason to focus on mood states or affective “tone”. While mood states or affective “tone” belong to somewhat different, but related, concepts compared with emotional responses, there is a large body of evidence showing that general mood states are of paramount importance for wellbeing, mental health and physical health, motivation, and cognition [17,18,19]. According to the broaden-and-build theory, a positive mood allows individuals to enter a broadened state of mind, which fuels the build-up of new personal resources [19].

### 1.4. Aims of the Present Study

The present study examined ambulatory HR and HRV assessed with an ECG, affective states assessed with ESM, and body position and physical activity assessed with accelerometry worn on the participants’ chests over seven days. A key aim was to explore avenues that bypass the above-mentioned challenges regarding changing body positions and activity patterns over time. In particular, we investigated the following research questions:

1. How do the HR and HRV measures differ between different body positions (i.e., posture) or current activity class (i.e., inactive vs. active) in ambulatory assessments?
2. Is the intensity of physical activity (measured as the metabolic equivalent of task; MET) associated with HR and HRV during ambulatory assessments?
3. Are the HR and HRV measures associated with positive and negative affect on a within-individual level?
4. Do the associations of research question 3 change in terms of significance and effect size if different time intervals for HRV sampling are considered (e.g., how do the associations change if HRV is measured over 5, 10, or 30 min)?
5. Do the associations of research question 3 change in terms of significance and effect size if body position and activity status are controlled statistically compared with when they are held constant by stratification (e.g., by particularly focusing on resting states in the upright body position)?
6. Do the associations of research question 3 change in terms of significance and effect size if single affect items (e.g., being happy, being sad, or being enthusiastic) are used instead of affect sum scores (i.e., positive and negative affect).
7. Are the HR and HRV measures associated with positive and negative affect on a between-individual level?

## 2. Materials and Methods

### 2.1. Participants

The sample in the present study was a subsample of a larger, four-week-long experience sampling study. Participants were recruited from a convenience sample through a variety of channels, including the study management portal of the Department of Psychology at Bielefeld University, email distribution lists of various German universities to which people can opt-in to receive information about studies that are recruiting participants, social media, and word of mouth. The participants signed up for the study via an online questionnaire in which they were informed about the procedure and conditions of the study, including data handling and protection. All participants gave informed consent. To participate in this study, the participants had to be 18 to 29 years old and had to have a smartphone with an Android operating system available for the duration of the study. If they did not have a smartphone with an Android operating system, they could use an Android lab phone during their participation.

**Figure 1:**
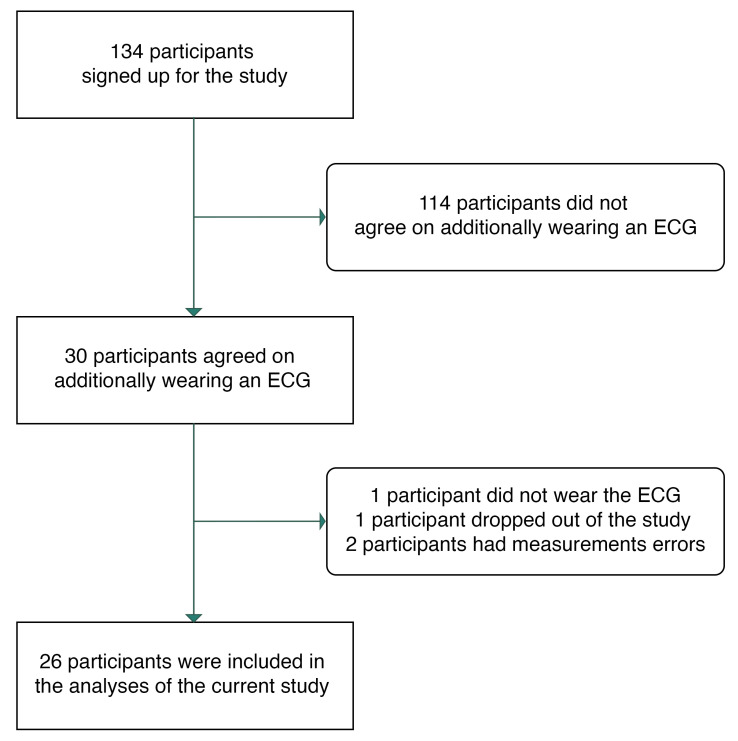
Participant inclusion flowchart.

As part of the larger study, 30 participants agreed to additionally wear an ECG device for one week during data collection. Four participants were not included in the present sample. One participant did not wear the ECG device and another one dropped out before data collection started. Two participants were excluded because of measurement errors, showing zero variation in ECG measures across the week. The inclusion and exclusion of participants are depicted in Figure 1. The final sample consisted of 26 generally healthy participants (23 females) with a mean age of 23.8 years (*SD* = 3.0 years; range: 19–29 years). The mean body mass index (BMI) was 22.3 kg/m^2^ (*SD* = 4.0 kg/m^2^; range: 18–34 kg/m^2^).

### 2.2. Procedure

The study was conducted in accordance with the Declaration of Helsinki and with the approval of the Ethics Committee of Bielefeld University (file no. 2022-063). Data were collected in two batches: batch 1 was collected from 26 April to 23 May 2022 and batch 2 was collected from 17th June to 15th July 2022. Each batch collection period lasted for 29 days.

On the first day of the study, participants completed a baseline questionnaire, in which demographic information was collected. On the second day, the ESM questionnaires presented on movisensXS started (version 1.5.23; library version 7450; movisens GmbH, Karlsruhe, Germany). Participants received notifications to fill out short questionnaires (approx. 2 min) seven times per day for 28 consecutive days. The participants could self-initiate the first questionnaire of a day manually. If they did not start the questionnaire manually, prompts for the first questionnaire were sent out at a random time between 8:00 a.m. and 9:00 a.m. on weekdays and between 9:00 a.m. and 10:00 a.m. on weekends. The prompts for the subsequent five questionnaires were sent out at random times between 10:30 a.m. and 7:30 p.m. on all days with an interval of at least 90 min between adjacent questionnaires. For the last questionnaire of a day, the prompts were sent out between 9:00 p.m. and 10:00 p.m. on all days. After receiving a prompt, the participants could respond to the questionnaire within 30 min. During that period, the participants received up to three more notifications to fill out the questionnaire if they had not yet completed or dismissed the respective questionnaire. If the participants did not respond to a questionnaire within 30 min or if they dismissed it, this questionnaire was considered as missing. The participants were instructed to ignore prompts in situations that could cause danger to themselves or others (e.g., while driving). The participants wore an ecgMove4 (movisens GmbH, Karlsruhe, Germany) for one week during their participation. The ecgMove4 includes both an accelerometer to measure physical activity and an ECG to measure cardiac activity. The participants were instructed to put on the ecgMove4 around their chest in the morning after getting up and to take it off in the evening before going to bed. We only used the ESM data of the week in which an ECG device was worn in the analyses.

The participants who completed at least 80% of all questionnaires received either a 90€ voucher or a 50€ voucher and research participation credits. The participants who completed between 50% and 80% of all questionnaires received half of the compensation. In addition, three 100€ vouchers were raffled among all participants who completed at least 50% of all questionnaires. The participants who wore an ECG device received an additional 30€ voucher.

### 2.3. Measures and Instruments

#### 2.3.1. Physical Activity Measures

The physical activity measures were derived from triaxial acceleration recorded at a sampling rate of 64 Hz with the ecgMove4. The raw acceleration data were processed with DataAnalyzer (Version 1.13.5; movisens GmbH, Karlsruhe, Germany) to obtain measures of body position, activity class, and energy expenditure in 1 min epochs. These variables were used to control for the effects of posture and current activity on the ECG measures.

The body position in a 1 min epoch was classified by using the mean inclination of the body axes derived from their acceleration signal [20]. The sensors worn on the chest could differentiate the body positions of lying supine, lying left, lying prone, lying right, and upright. If the body position could not be classified for a 1 min epoch, it was classified as unknown. For further analysis, all lying positions were combined into one, leaving a total of three categories: unknown, lying, and upright.

The activity in a 1 min epoch was classified by an algorithm using the acceleration signal and barometric air pressure [21,22]. The sensors worn on the chest could detect the activity classes lying, sitting/standing, cycling, jogging, and walking. If the body position of a 1 min epoch was classified as unknown, the activity class for the same epoch was considered as unknown as well. For further analysis, the activity classes of lying and sitting/standing were combined into inactive and cycling, jogging, and walking into active.

The MET was used as a measure of the intensity of physical activity. METs are the ratio of the resting metabolic rate and represent the metabolic rate during physical activity [23]. The MET calculation is based on personal parameters (age, gender, weight, and height), as well as the activity class and bandpass-filtered movement acceleration for the corresponding 1 min epoch [24].

For further analyses, the body position and activity class were aggregated for (1) different time intervals (5, 10, and 30 min) before the participants responded to each ESM questionnaire, (2) all measurements recorded on the same day before an ESM questionnaire, and (3) all measurements recorded on the same day after an ESM questionnaire. For each time interval, the proportions of the time during which the participants were upright (body position) and active (activity class) were calculated. The 1 min epochs with unknown body positions or activity classes were excluded.

#### 2.3.2. Cardiac Measures

The ecgMove4 recorded ECG data with a sampling rate of 1024 Hz. The raw ECG data were processed with DataAnalyzer (Version 1.13.5; movisens GmbH, Karlsruhe, Germany) to obtain the HR- and HRV-related variables in 1 min epochs. The low-frequency power (HRV-LF), high-frequency power (HRV-HF), ratio of low to high-frequency power (LF/HF ratio), and root-mean-square of successive differences (RMSSD) were extracted. Measures in the frequency domain were derived by using fast Fourier transformation following Welch’s method [25]. The processing of the ECG data included artefact detection and rejection, R-peak identification, NN list generation, segmentation, and detrending. The processing steps and the algorithm used to calculate the HRV outcomes are described in the movisens online documentation [26]. These processing steps and algorithms also determined the validity of the HRV measurements in each 1 min epoch. The proportions of valid and invalid epochs in different body positions and activity classes are displayed in Table 1. All epochs with invalid HRV measurements were excluded from further processing.

For further analyses, the HR and HRV measures were aggregated into mean values of (1) different time intervals (5, 10, and 30 min) before the participants answered each ESM questionnaire, (2) all measurements recorded on the same day before the questionnaire, and (3) all measurements recorded on the same day after the questionnaire.

#### 2.3.3. Affective States

The assessment of affective states was based on Das-Friebel et al. [27], but there were some modifications to include fewer items measuring positive and negative affect. Positive affect was measured with three items, including content, enthusiastic, and happy. Negative affect was measured with three items, including sad, upset, and worried. These items were originally taken from the Positive and Negative Affect Scale [28] and Russell’s Circumplex Model of Affect [29]. In each of the ESM questionnaires, the participants were asked to indicate on a visual analogue scale how they felt in that moment (“How … do you feel at the moment?”, 0 = not at all, 100 = very much). Internal consistency was found to be good for positive affect (Cronbach’s α = 0.87) and negative affect (Cronbach’s α = 0.80). The sum scores for positive and negative affect were computed separately. Higher sum scores indicated higher levels of positive and negative affect.

### 2.4. Data Analyses

Data pre-processing and all statistical analyses were conducted in R (Version 4.2.1) [30]. For all analyses, the *p*-values were adjusted for multiple comparisons using the false discovery rate (FDR) [31], and the significance threshold was set to 0.05.

To answer research questions 1 to 6 on within-individual differences, we computed multilevel models using the R package lme4 (Version 1.1-30) [32]. All models were set up as random intercept models and maximum likelihood estimation was used. All continuous variables were within-individual standardised before the statistical analyses to account for within-individual effects and to facilitate interpretation. To test research question 1, the dichotomous variables body position (lying vs. upright) and activity class (inactive vs. active) were included as predictors for the HR and HRV measures in the models to test the differences between the respective categories. Research question 2 was tested by assigning the MET of 1 min epochs as a predictor for the HR and HRV measures of the same epoch. Research questions 3 and 4 were tested by aggregating the HR and HRV measures into different intervals (5, 10, and 30 min before answering an ESM questionnaire) as predictors for the positive and negative affective sum scores. A single model was computed for each combination of the predictor, time interval, and outcome. The proportions of time spent upright (body position) and active (activity class) in the respective time intervals were included as covariates. Furthermore, we repeated these analyses in a stratified sample (research question 5), in which only the measurements of an upright body position and inactivity for at least 80% of the time in the corresponding time intervals were included. In the stratified analysis, we additionally used the HR and HRV aggregates of all measurements recorded before and after the participants started answering the ESM questionnaire on a given day as predictors and compared them with the time intervals (5, 10, and 30 min) directly before the questionnaire. Moreover, we analysed single affect items as outcomes using the HR and HRV predictors of the 5 min time interval before the participants started an ESM questionnaire (research question 6).

For the between-individual analyses detailed in research question 7, the personal mean values of affective states and HR and HRV measures were calculated. For the HR and HRV measures, additional mean values were calculated by only including measurements in which the participant was (1) lying, (2) upright, (3) inactive, (4) active, or (5) upright and inactive. All variables were grand-mean-standardised before the analyses. Separate linear regressions were computed by assigning each participant’s mean HR and HRV measures as predictors (including the means stratified for activity class and body position) of the means of the affective state sum scores and single items. Age, gender, and BMI were included as covariates.

## 3. Results

### 3.1. Descriptive Statistics

**Figure 2:**
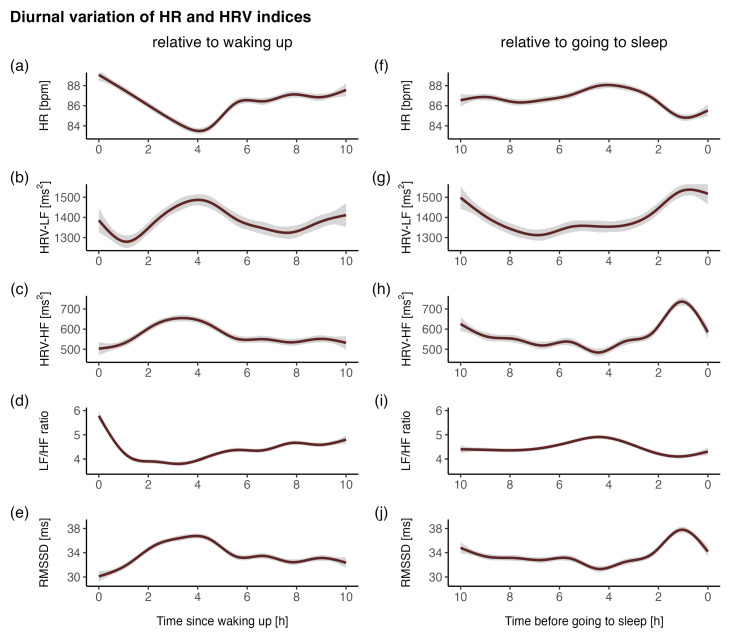
Diurnal variations in the HR and HRV indices (**a**–**e**) relative to waking up (start of wear time) and (**f**–**j**) relative to going to sleep (end of wear time). (**a**,**f**) HR, heart rate; (**b**,**g**) HRV-LF, low-frequency power; (**c**,**h**) HRV-HF, high-frequency power; (**d**,**i**) LF/HF ratio, ratio of low- to high-frequency power; (**e**,**j**) RMSSD, root-mean-square of successive differences. The indices were averaged across all individuals and days.

On average, the participants responded to questionnaires and provided corresponding ECG data in 72.7% of all possible cases, resulting in 926 assessments in total (on average, 35.6 per participant). For the ECG measurements alone, there were 130,040 valid 1 min epochs. Considering all available and valid epochs, the participants were mostly in an upright body position (83.0%; 15.6% lying and 1.4% unknown) and inactive (91.7%; 7.1% active and 1.2% unknown). In 76.1% of all epochs, the participants were in an upright body position and inactive at the same time. The demographics and overall descriptive statistics are presented in Table 2. The descriptive statistics stratified for different body positions and activity classes are displayed in Table 3. The diurnal variations in the HR and HRV indices are presented in Figure 2.

### 3.2. Differences in HR and HRV Measures between Body Positions and Activity Classes

When comparing HR and HRV measurements between body positions, it was shown that the HR (β = 0.80, *p* < 0.001), HRV-LF (β = 0.07, *p* < 0.001), and LF/HF ratio (β = 0.55, *p* < 0.001) were higher in an upright position than in a lying position, while HRV-HF (β = −0.30, *p* < 0.001) and RMSSD (β = −0.38, *p* < 0.001) were lower.

**Figure 3:**
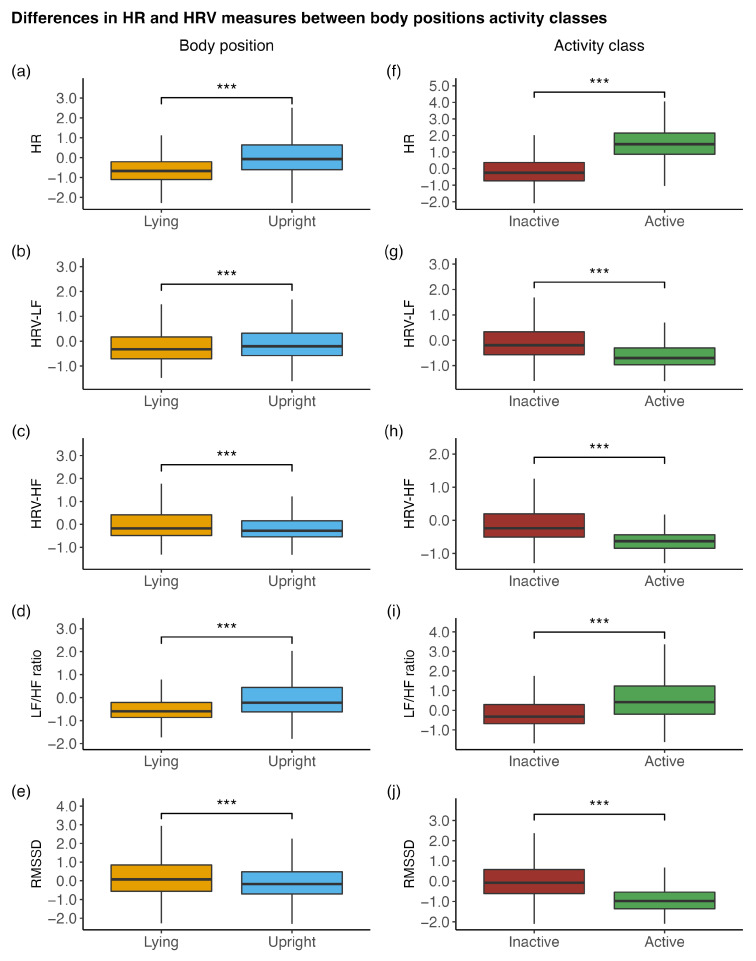
Differences in the HR and HRV measures between (**a**–**e**) body positions (lying vs. upright) and (**f**–**j**) activity classes (inactive vs. active). HR, heart rate; HRV-LF, low-frequency power; HRV-HF, high-frequency power; LF/HF ratio, ratio of low- to high-frequency power; RMSSD, root-mean-square of successive differences. All depicted HR and HRV measures were within-individual standardised. *** *p* < 0.001.

When comparing HR and HRV measurements between activity classes, it was found that the HR (β = 1.76, *p* < 0.001) and the LF/HF ratio (β = 0.80, *p* < 0.001) were higher during activity than during inactivity, while the HRV-LF (β = −0.59, *p* < 0.001), the HRV-HF (β = −0.60, *p* < 0.001), and the RMSSD (β = −0.98, *p* < 0.001) were lower. Due to within-individual standardisation, the β-values of these analyses can be interpreted similarly to a standardised mean difference between the respective conditions. All comparisons are displayed in Figure 3.

### 3.3. Within-Individual Associations of Physical Activity with HR and HRV

Multilevel models revealed that MET was associated with the HR (β = 0.56, *p* < 0.001), HRV-LF (β = −0.14, *p* < 0.001), HRV-HF (β = −0.18, *p* < 0.001), LF/HF ratio (β = 0.26, *p* < 0.001), and RMSSD (β = −0.29, *p* < 0.001).

### 3.4. Within-Individual Associations of HR and HRV with Affective Wellbeing

When analysing the ECG aggregates of the 5 min intervals before answering the questionnaire while solely statistically controlling for body position and activity class, the HR (β = 0.14, *p* < 0.01) and LF/HF ratio (β = 0.11, *p* < 0.05) were positively associated with positive affect, while the other measures (HRV-LF, HRV-HF, and RMSSD) were unrelated. The results were relatively consistent in terms of effect size and significance when different time intervals of aggregated ECG measures (i.e., 10 and 30 min) were considered. No associations between HRV measures and negative affect were found. All associations are displayed in Table 4.

When analysing these associations stratified for being in an upright body position and inactive at the same time under consideration of the ECG aggregates of the 5 min interval before starting the questionnaire, the results appeared to be more pronounced compared with only controlling for body position and activity class. The HR (β = 0.15, *p* < 0.01) and LF/HF ratio (β = 0.14, *p* < 0.01) were associated with higher positive affect. The HRV-HF (β = −0.11, *p* < 0.05), and RMSSD (β = −0.11, *p* < 0.05) were associated with lower positive affect. No association was found for HRV-LF.

The effect sizes of the associations of HR (β = 0.19, *p* < 0.001), HRV-HF (β = −0.13, *p* < 0.05), (β = 0.13, *p* < 0.05), and RMSSD (β = −0.13, *p* < 0.05) with positive affect were similar when analysing the ECG aggregates of the 10 min intervals before starting the questionnaire. For the aggregates of the 30 min intervals and of all continuous measures of a day before starting the questionnaire, only the association of HR and LF/HF ratio with positive affect remained significant. No significant association was found for any HR or HRV measure when their aggregates were based on all remaining measures in a sampling day after starting the questionnaire. Similarly, no associations with negative affect were found for any of the measures, regardless of the different time intervals for which the measures were aggregated. All associations are displayed in Table 5.

Item-level analyses revealed that the effects of the HR and LF/HF ratio were driven by the associations with the item “enthusiastic” and, to a somewhat lesser degree, by “happy”. Being enthusiastic was associated with the HR (β = 0.19, *p* < 0.001), HRV-HF (β = −0.12, *p* < 0.05), LF/HF ratio (β = 0.14, *p* < 0.01), and RMSSD (β = −0.14, *p* < 0.01). Being happy was associated with the HR (β = 0.12, *p* < 0.05) and LF/HF ratio (β = 0.12, *p* < 0.05). Furthermore, a higher LF/HF ratio was followed by being less worried (β = −0.13, *p* < 0.05). Other affect items were unrelated to the HR and HRV measures. All associations are displayed in Table 6.

### 3.5. Between-Individual Associations of HR and HRV with Affective Wellbeing

**Figure 4:**
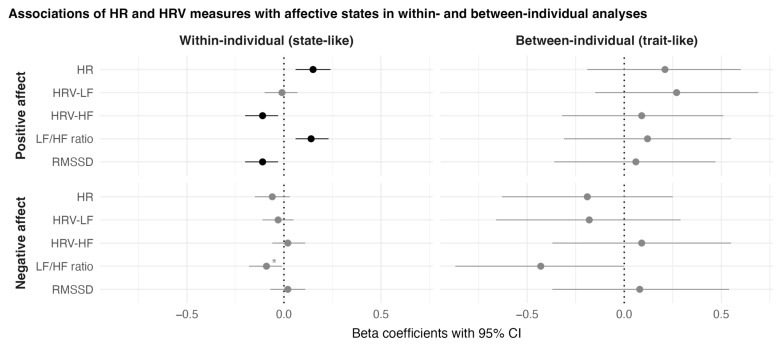
Results of within-individual and between-individual analyses of the associations between HR and HRV measures and affective states stratified for being in an upright body position and being inactive. HR, heart rate; HRV-LF, low-frequency power; HRV-HF, high-frequency power; LF/HF ratio, ratio of low- to high-frequency power; RMSSD, root-mean-square of successive differences; CI, confidence interval. The depicted confidence intervals have not been corrected for multiple comparisons. Interpretations concerning the significance of an association might, therefore, differ from the FDR-corrected *p*-values. Black coloured dots and Cis indicate associations that were significant after FDR correction. Asterisks (*) indicate associations that were significant before, but not after, FDR correction.

The linear regressions revealed no significant between-individual associations of the HR and HRV measures with positive or negative affect. All associations are displayed in Table 7. The results of the within- and between-individual analyses are presented in Figure 4.

## 4. Discussion

The aim of this study was to investigate the relationship of the HR and HRV measures with affective states in everyday life and to present an approach that would overcome the challenges associated with ambulatory assessments of HRV, physical activity, and body position in combination with ESM. We, therefore, applied ambulatory ECG measurements in combination with an ESM design across seven consecutive days by additionally considering body posture and physical activity.

Moreover, we attempted to answer seven questions. First, we asked how the HR and HRV measures would differ between different body positions or current activity classes. We found that the HR and HRV measures significantly differed between body positions (lying vs. upright) and activity classes (inactive vs. active). Second, we asked how the intensity of physical activity (measured in MET) would relate to the HR and HRV measures. We found that the intensity of physical activity was accompanied by a higher HR and LF/HF ratio and by lower HRV-LF, HRV-HF, and RMSSD. Third, we asked how the HR and HRV measures would relate to positive and negative affect on a within-individual level and, fourth, how these associations would change if different time intervals of HRV sampling were considered (e.g., 5, 10, or 30 min). We found that a higher HR and LF/HF ratio in different time intervals before a questionnaire assessment were associated with higher levels of positive affect. Furthermore, HRV-HF and RMSSD were negatively associated with positive affect, but these were only found when aggregates of shorter time intervals (5 and 10 min) before a questionnaire assessment were considered. No associations with negative affect were found. Fifth, we asked how the associations of HR and HRV measures with positive and negative affect would change if they were analysed while stratified for an upright body position and being inactive. We found that the associations were more pronounced compared with the analyses in which we only statistically controlled for body position and activity class. Sixth, we asked how HR and HRV measures would be related to single affect items in contrast to affect sum scores. We found that the associations of HR and HRV measures with positive affect were mainly driven by the associations with the item of feeling enthusiastic and, to a lesser degree, the item of feeling happy. Seventh, we asked how the HR and HRV measures relate to positive and negative affect on a between-individual level. We did not find that any HR or HRV measures were related to positive or negative affect on a between-individual level.

Our finding that HR and HRV measures differed between body positions and activity classes and that they were related to physical activity measured in MET is in line with the recommendations made by the Task Force [7] and with findings by another study that applied ambulatory assessments of HRV and investigated the influences of body position and activity [14]. These findings emphasize the importance of considering the current body position and activity when investigating the associations of HR and HRV measures with other measures, such as affective states. In this study, we focused on studying “tonic” HR and HRV measures, specifically while being inactive and in an upright position. To take a step further, future studies that combine ESM and ambulatory ECG assessments could investigate segments of “reactivity” (i.e., a sudden increase in physical activity/metabolic activity) and “recovery” (i.e., a period of inactivity following vigorous activity) and their associations with different outcome measures [8,33]. Furthermore, future studies could include scheduled tests, such as the orthostatic test, to examine repeatedly and in a standardised way the ability of the autonomic function to react to a change in body position [33].

In line with laboratory-based findings by Shi et al. [9], we found that a higher LF/HF ratio was consistently associated with higher positive affect, specifically with feeling enthusiastic and happy. A higher LF/HF ratio is generally considered to represent SNS dominance over the PNS [4,5]. This suggests that higher SNS activity relates to improved affective wellbeing. In accordance, in a stratified analysis focusing only on the upright body position and the inactive state, we found that HRV-HF and RMSSD, which represent PNS activity [4,5], were negatively associated with positive affect and, specifically, with feeling enthusiastic. This is in line with Schwerdtfeger and Gerteis [14], who also found a negative within-individual association between positive affect (e.g., feeling enthusiastic) and RMSSD. Furthermore, we showed that physical activity operationalised as MET was accompanied by SNS dominance (higher LF/HF ratio, lower HRV-HF, and RMSSD). Prior research applying ESM found that physical activity also has acute beneficial effects on affective wellbeing [34,35,36,37,38]. Based on this notion, SNS activation could be a mediating factor of the effect of physical activity on affective wellbeing, specifically higher positive affect. However, this notion, as well as our findings that SNS dominance was associated with higher levels of positive affect, are in contrast to the findings of Kok and Fredrickson [39], who found that a higher parasympathetic (vagal) tone indicated by respiratory sinus arrhythmia was associated with experiencing more positive emotions. Referring to the broaden-and-build theory [19], they posited that the HRV and emotions interact in such a way that a higher parasympathetic tone enhances the experience of positive emotions, which, in turn, would facilitate the building up of personal resources. The apparent contradiction between our findings and the study by Kok and Fredrickson [39] could possibly be explained by the differences in study designs and, particularly, whether between-individual/trait-level or within-individual/state-level data are analysed. Kok and Fredrickson [39] analysed between-individual variance (i.e., trait-level data). When we analysed between-individual variance, we could not find an association. However, we found an association that was in the opposite direction when we analysed associations based on within-individual variance. It is conceivable, for instance, that higher general physical fitness levels positively contribute to positive emotions, as well as to higher trait-level parasympathetic tone. In contrast, considering within-individual variation, it is also conceivable that, in states with very low arousal, people may experience less positive affect than when arousal is on a somewhat higher and intermediate level, which may particularly apply to the experience of high-arousal positive emotions, such as enthusiasm. A similar notion is described by the Yerkes–Dodson law [40], namely that performance levels show an inverted U-shaped association with activation levels. Performance is best at an intermediate level of activation, while performance decreases if activation is too low or too high. It is possible that, in our participants’ everyday lives, an increase in activation mostly facilitates them entering a more ideal “activation zone” and, therefore, possibly increases positive affect, as their average activation level is possibly somewhat lower than their ideal activation level.

Apart from the level of activation, the general pleasantness of the situation may play a role, too. In our study, participants were prompted at random times in their everyday life, and their mood was, on average, rather favourable, but not overly activated. This possibly is in sharp contrast to studies in which individuals are examined in laboratory experiments when HRV and emotional experiences are measured in response to pleasant and unpleasant stimuli. It is conceivable that, for instance, being exposed to unpleasant pictures or watching frightening film clips in a laboratory experiment [41] may evoke more intense affective responses. In a similar way, it is conceivable that, in unpleasant laboratory situations, individuals can easily be pushed beyond the “zone of ideal activation” and that they may experience intense negative emotions due to entering a state of sympathetic hyperarousal.

It is also worth noting that the average resting HR (referring to epochs in which participants were upright and inactive) in our study was higher than that which would be expected [42]. Various aspects can be relevant in this regard. For example, the activity status of the participants before the corresponding resting epochs is not taken into account. For example, the participants could have exercised before, which could lead to an elevated HR in subsequent resting epochs. Moreover, we could not distinguish between the upright sitting and upright standing positions, nor could we exclude epochs during which participants were under the influence of caffeine. Relatedly, a higher HR was also observed in the sample of a study by Kim et al. [15], who followed a similar research approach and performed ECG measurement with the Polar H10 (Polar Electro Oy, Kempele, Finland), which has been described as the current gold standard of ambulatory ECG measurement [43].

### 4.1. Strengths and Limitations

To the best of our knowledge, this is the first study to investigate the associations of multiple HR and HRV measures with positive and negative affective states, including single affective item analyses, by applying ambulatory ECG assessments in the context of ESM.

However, this study is not without limitations. First, our sample was not representative of the general population and included mainly young female university students. Gender differences could play a role, as with many other psychological constructs and biological processes. Second, we adopted an observational design that does not allow any causal inferences to be drawn. Third, we did not take into account further behavioural and contextual factors (e.g., the consumption of caffeine or nicotine before filling out the questionnaires) that may change HR and HRV and influence on positive and negative affect (see, e.g., Schwerdtfeger et al. [14] for a discussion of potentially influential factors). Fourth, the participants were inactive and in an upright position during most of the sampling periods. Therefore, conclusions can only be drawn regarding the investigated relationships for an upright posture and inactivity. In this regard, based on the available accelerometry data, it was not possible to differentiate whether the participants were sitting or standing when they were in an upright position. Fifth, the relatively small sample size had insufficient power for the between-subject analyses. Sixth, the current study does not consider any non-linear HRV indices or techniques (e.g., sliding trend fuzzy approximate entropy, information-based similarity index, or temporal dependency complexity analysis) that may inform further important aspects of the HRV. Future studies may also include such non-linear HRV indices.

### 4.2. Conclusions

This study provides unique insights into the relationships of various ambulatory assessed HRV measures, including the HRV-LF, HRV-HF, LF/HF ratio, and RMSSD, with positive and negative affective states in everyday life. In conclusion, we found small, but stable, within-individual associations of the HR and LF/HF ratio with positive affect, specifically in being enthusiastic and happy, which implies that a relative activation of the SNS compared with the PNS and/or PNS withdrawal has beneficial effects on positive affect. No associations were found with negative affect for any HR and HRV measures.

## Figures and Tables

**Table 1:** Proportions of invalid vs. valid epochs of HR and HRV measurements depending on body position and activity class.

		HR and HRV Measurements	
		Invalid	Valid
Body position	Lying	9.5%	90.5%
	Upright	6.9%	93.1%
Activity class	Inactive	5.7%	94.3%
	Active	25.1%	74.9%

**Table 2:** Demographics and descriptive statistics.

		Full Sample *N* = 26
		*n* (%)/*M* (*SD*)
*Demographics*	Gender	
	Female	23 (88.5)
	Male	3 (11.5)
	Age	23.8 (3.0)
	Weight (kg)	66.1 (13.1)
	Height (cm)	171.7 (8.3)
	BMI (kg/m^2^)	22.3 (4.0)
*ECG and accelerometry*	MET	1.5 (0.1)
	HR (bpm)	87.3 (8.6)
	HRV-LF (ms^2^)	1303.2 (667.5)
	HRV-HF (ms^2^)	590.3 (389.9)
	LF/HF ratio	4.2 (1.5)
	RMSSD (ms)	33.6 (12.8)
*Experience sampling*	Questionnaires available	926
	per day	132.3 (5.4)
	per participant	35.6 (5.8)
	per day	5.1 (1.2)
	Positive affect	172.0 (43.0)
	Content	61.4 (15.2)
	Enthusiastic	49.7 (15.1)
	Happy	61.0 (15.2)
	Negative affect	60.8 (46.1)
	Sad	19.0 (16.9)
	Upset	13.5 (12.2)
	Worried	28.2 (20.3)

BMI, body mass index; ECG, electrocardiogram; MET, metabolic equivalent of task; HR, heart rate; HRV-LF, low-frequency power of heart rate variability; HRV-HF, high-frequency power of heart rate variability; RMSSD, root-mean-square of successive differences.

**Table 3:** Descriptive statistics for HR/HRV measures stratified for body position and activity class.

	BP = Lying (*n* = 20,235)	BP = Upright (*n* = 107,989)	AC = Inactive (*n* = 119,185)	AC = Active (*n* = 9263)	BP = Upright/ AC = Inactive (*n* = 98,968)
	*M* (*SD*)	*M* (*SD*)	*M* (*SD*)	*M* (*SD*)	*M* (*SD*)
Epochs per participant	778.3 (704.3)	4153.4 (952.0)	4584.0 (890.2)	356.3 (229.1)	3806.5 (868.8)
HR (bpm)	78.9 (11.2)	89.0 (14.5)	85.3 (12.8)	112.4 (13.8)	86.8 (12.6)
HRV-LF (ms^2^)	1424.6 (1247.6)	1310.1 (1192.0)	1360.3 (1212.6)	678.2 (713.6)	1375.0 (1199.7)
HRV-HF (ms^2^)	996.4 (968.2)	548.8 (638.7)	624.9 (709.0)	194.6 (338.4)	583.6 (646.0)
LF/HF ratio	2.7 (2.1)	4.5 (3.5)	4.0 (3.2)	6.7 (4.2)	4.3 (3.3)
RMSSD (ms)	43.5 (20.0)	32.4 (16.3)	34.9 (17.1)	17.6 (11.3)	33.8 (15.9)

BP, body position; AC, activity class; *n,* number of available epochs for all participants; HR, heart rate; HRV-LF, low-frequency power of heart rate variability; HRV-HF, high-frequency power of heart rate variability; RMSSD, root-mean-square of successive differences.

**Table 4:** Within-individual associations of HR and HRV parameters in different epoch lengths before filling out the ESM questionnaires with positive and negative affect sum scores controlling for body position and activity class.

		Outcome										
		Positive Affect						Negative Affect				
Aggregate	Predictor	β	*SE*	*df*	*t*	*p*		β	*SE*	*df*	*t*	*p*
5 min	HR	0.14	0.04	882	3.40	<0.01		−0.03	0.04	882	−0.75	0.729
	HRV-LF	0.01	0.04	855	0.18	0.906		−0.05	0.04	855	−1.27	0.476
	HRV-HF	−0.03	0.04	855	−0.73	0.729		−0.02	0.04	855	−0.60	0.778
	LF/HF ratio	0.11	0.04	855	3.13	<0.05		−0.07	0.04	855	−2.01	0.173
	RMSSD	−0.04	0.04	862	−0.99	0.625		−0.03	0.04	862	−0.71	0.733
												
10 min	HR	0.16	0.04	894	4.09	<0.001		−0.01	0.04	894	−0.29	0.875
	HRV-LF	−0.02	0.04	872	−0.54	0.778		−0.02	0.04	872	−0.69	0.751
	HRV-HF	−0.07	0.03	872	−1.89	0.209		−0.03	0.04	872	−0.72	0.733
	LF/HF ratio	0.11	0.04	872	3.11	<0.05		−0.03	0.04	872	−0.76	0.729
	RMSSD	−0.07	0.04	879	−1.96	0.185		−0.02	0.04	879	−0.46	0.814
												
30 min	HR	0.15	0.04	916	3.90	<0.01		−0.02	0.04	916	−0.53	0.778
	HRV-LF	0.00	0.03	899	0.03	0.990		−0.03	0.03	899	−0.84	0.700
	HRV-HF	−0.04	0.03	899	−1.15	0.547		0.00	0.03	899	−0.12	0.929
	LF/HF ratio	0.12	0.04	899	3.32	<0.01		−0.05	0.04	899	−1.37	0.427
	RMSSD	−0.06	0.04	904	−1.69	0.283		0.00	0.04	904	−0.12	0.929

HR, heart rate; HRV-LF, low-frequency power of heart rate variability; HRV-HF, high-frequency power of heart rate variability; RMSSD, root-mean-square of successive differences. All *p*-values represent the values after FDR correction.

**Table 5:** Within-individual associations of HR and HRV parameters in different epoch lengths before or after filling out the ESM questionnaires with positive and negative affect sum scores stratified for being in an upright body position and being inactive.

		Outcome										
		Positive Affect						Negative Affect				
Aggregate	Predictor	β	*SE*	*df*	*t*	*p*		β	*SE*	*df*	*t*	*p*
5 min	HR	0.15	0.04	643	3.37	<0.01		−0.06	0.04	643	−1.38	0.427
	HRV-LF	−0.01	0.04	619	−0.36	0.840		−0.03	0.04	619	−0.77	0.729
	HRV-HF	−0.11	0.04	619	−2.61	<0.05		0.02	0.04	619	0.57	0.778
	LF/HF ratio	0.14	0.04	619	3.35	<0.01		−0.09	0.04	619	−2.14	0.144
	RMSSD	−0.11	0.04	625	−2.62	<0.05		0.02	0.04	625	0.41	0.827
												
10 min	HR	0.19	0.04	632	4.27	<0.001		−0.06	0.04	632	−1.33	0.452
	HRV-LF	−0.04	0.04	613	−0.95	0.653		−0.02	0.04	613	−0.47	0.814
	HRV-HF	−0.13	0.04	613	−3.03	<0.05		0.01	0.04	613	0.17	0.910
	LF/HF ratio	0.13	0.04	613	3.13	<0.05		−0.05	0.04	613	−1.17	0.532
	RMSSD	−0.13	0.04	620	−2.93	<0.05		0.02	0.04	620	0.56	0.778
												
30 min	HR	0.16	0.04	622	3.64	<0.01		−0.01	0.05	622	−0.31	0.870
	HRV-LF	−0.02	0.04	605	−0.54	0.778		−0.04	0.04	605	−0.93	0.664
	HRV-HF	−0.08	0.05	605	−1.72	0.273		0.00	0.05	605	−0.09	0.950
	LF/HF ratio	0.13	0.04	605	3.01	<0.05		−0.03	0.04	605	−0.61	0.778
	RMSSD	−0.09	0.04	610	−2.02	0.173		−0.02	0.05	610	−0.34	0.846
												
Before ^a^	HR	0.12	0.04	652	2.94	<0.05		−0.08	0.04	652	−1.89	0.209
	HRV-LF	−0.04	0.04	646	−1.04	0.607		−0.03	0.04	646	−0.62	0.778
	HRV-HF	−0.06	0.04	646	−1.57	0.339		0.00	0.04	646	−0.03	0.990
	LF/HF ratio	0.11	0.04	646	2.82	<0.05		−0.08	0.04	646	−2.09	0.155
	RMSSD	−0.06	0.04	648	−1.45	0.405		0.01	0.04	648	0.16	0.914
												
After ^b^	HR	0.06	0.05	540	1.21	0.503		−0.01	0.04	540	−0.19	0.906
	HRV-LF	0.04	0.04	539	0.87	0.684		−0.09	0.04	539	−2.10	0.155
	HRV-HF	0.01	0.05	539	0.21	0.906		−0.05	0.04	539	−1.24	0.494
	LF/HF ratio	0.02	0.05	539	0.46	0.814		0.02	0.04	539	0.38	0.840
	RMSSD	0.00	0.04	539	−0.01	0.995		−0.03	0.04	539	−0.67	0.765

HR, heart rate; HRV-LF, low-frequency power; HRV-HF, high-frequency power; RMSSD, root-mean-square of successive differences. All *p*-values represent the values after FDR correction. ^a^ aggregates of all continuous measures of a day before filling out the questionnaire. ^b^ aggregates of all continuous measures of a day after filling out the questionnaire.

**Table 6:** Within-individual associations of HR and HRV parameters with an epoch length of 5 min before filling out ESM questionnaires with single affect items stratified for being in an upright body position and being inactive

	Outcome	Predictor	β	*SE*	*df*	*t*	*p*
Positive affect	Content	HR	0.06	0.04	643	1.38	0.427
		HRV-LF	−0.02	0.04	619	−0.59	0.778
		HRV-HF	−0.07	0.04	619	−1.63	0.312
		LF/HF ratio	0.08	0.04	619	1.79	0.239
		RMSSD	−0.06	0.04	625	−1.43	0.409
							
	Enthusiastic	HR	0.19	0.04	643	4.25	**<0.001**
		HRV-LF	−0.04	0.04	619	−0.99	0.625
		HRV-HF	−0.12	0.04	619	−2.85	**<0.05**
		LF/HF ratio	0.14	0.04	619	3.39	**<0.01**
		RMSSD	−0.14	0.04	625	−3.23	**<0.01**
							
	Happy	HR	0.12	0.04	643	2.76	**<0.05**
		HRV-LF	0.06	0.04	619	1.37	0.427
		HRV-HF	−0.04	0.04	619	−1.03	0.607
		LF/HF ratio	0.12	0.04	619	2.94	**<0.05**
		RMSSD	−0.04	0.04	625	−0.89	0.680
							
Negative affect	Sad	HR	−0.11	0.04	643	−2.42	0.070
		HRV-LF	0.01	0.04	619	0.18	0.906
		HRV-HF	0.05	0.04	619	1.07	0.589
		LF/HF ratio	−0.08	0.04	619	−1.86	0.217
		RMSSD	0.07	0.04	625	1.64	0.309
							
	Upset	HR	0.03	0.05	643	0.57	0.778
		HRV-LF	−0.01	0.04	619	−0.20	0.906
		HRV-HF	−0.03	0.04	619	−0.77	0.729
		LF/HF ratio	0.02	0.04	619	0.41	0.827
		RMSSD	−0.05	0.04	625	−1.12	0.562
							
	Worried	HR	−0.08	0.04	643	−1.84	0.222
		HRV-LF	−0.04	0.04	619	−1.07	0.589
		HRV-HF	0.06	0.04	619	1.43	0.409
		LF/HF ratio	−0.13	0.04	619	−3.02	**<0.05**
		RMSSD	0.07	0.04	625	1.60	0.322

HR, heart rate; HRV-LF, low-frequency power; HRV-HF, high-frequency power; RMSSD, root-mean-square of successive differences. All *p*-values represent values after FDR correction.

**Table 7:** Between-individual associations of HR and HRV with positive and negative affect sum scores.

		Outcomes										
		Positive Affect						Negative Affect				
Stratification	Predictor	β	*SE*	*df*	*t*	*p*		β	*SE*	*df*	*t*	*p*
Overall	HR	0.18	0.19	26	0.94	0.665		−0.16	0.21	26	−0.76	0.729
	HRV-LF	0.28	0.20	26	1.41	0.430		−0.19	0.23	26	−0.85	0.700
	HRV-HF	0.15	0.20	26	0.76	0.729		0.04	0.22	26	0.19	0.906
	LF/HF ratio	0.14	0.21	26	0.65	0.778		−0.46	0.21	26	−2.18	0.169
	RMSSD	0.10	0.20	26	0.52	0.786		0.06	0.22	26	0.26	0.882
												
BP = lying	HR	−0.06	0.19	26	−0.29	0.875		0.11	0.21	26	0.50	0.797
	HRV-LF	0.35	0.18	26	1.91	0.231		−0.19	0.21	26	−0.89	0.684
	HRV-HF	0.30	0.19	26	1.61	0.349		−0.06	0.22	26	−0.27	0.877
	LF/HF ratio	0.11	0.20	26	0.55	0.778		−0.28	0.22	26	−1.32	0.476
	RMSSD	0.30	0.19	26	1.60	0.350		−0.12	0.22	26	−0.55	0.778
												
BP = upright	HR	0.21	0.19	26	1.13	0.575		−0.21	0.21	26	−1.02	0.625
	HRV-LF	0.30	0.20	26	1.48	0.409		−0.20	0.23	26	−0.86	0.700
	HRV-HF	0.11	0.20	26	0.54	0.778		0.08	0.22	26	0.37	0.840
	LF/HF ratio	0.12	0.21	26	0.59	0.778		−0.45	0.21	26	−2.14	0.173
	RMSSD	0.07	0.20	26	0.35	0.846		0.08	0.22	26	0.38	0.840
												
AC = inactive	HR	0.18	0.19	26	0.97	0.653		−0.16	0.21	26	−0.74	0.729
	HRV-LF	0.25	0.20	26	1.26	0.498		−0.17	0.22	26	−0.75	0.729
	HRV-HF	0.13	0.20	26	0.65	0.778		0.05	0.22	26	0.24	0.891
	LF/HF ratio	0.13	0.21	26	0.64	0.778		−0.44	0.21	26	−2.11	0.179
	RMSSD	0.09	0.20	26	0.43	0.826		0.07	0.22	26	0.30	0.875
												
AC = active	HR	0.12	0.20	26	0.60	0.778		−0.10	0.22	26	−0.44	0.826
	HRV-LF	0.40	0.22	26	1.77	0.283		−0.33	0.26	26	−1.29	0.489
	HRV-HF	0.18	0.20	26	0.94	0.666		0.00	0.22	26	0.01	0.995
	LF/HF ratio	−0.11	0.20	26	−0.55	0.778		−0.05	0.22	26	−0.23	0.895
	RMSSD	0.17	0.21	26	0.84	0.700		0.03	0.23	26	0.14	0.928
												
BP = upright/	HR	0.21	0.19	26	1.10	0.589		−0.19	0.21	26	−0.91	0.680
AC = inactive	HRV-LF	0.27	0.20	26	1.36	0.455		−0.18	0.23	26	−0.81	0.727
	HRV-HF	0.09	0.20	26	0.46	0.814		0.09	0.22	26	0.40	0.840
	LF/HF ratio	0.12	0.21	26	0.59	0.778		−0.43	0.21	26	−2.07	0.185
	RMSSD	0.06	0.20	26	0.28	0.875		0.08	0.22	26	0.39	0.840

HR, heart rate; HRV-LF, low-frequency power; HRV-HF, high-frequency power; RMSSD, root mean square of successive differences. All *p*-values represent the values after FDR correction.

## Data Availability

The data presented in this study are available on request from the corresponding author. The data are not publicly available due to privacy or ethical restrictions.

## References

[1] Kreibig S.D. (2010). Autonomic Nervous System Activity in Emotion: A Review. Biol. Psychol..

[2] Pham T., Lau Z.J., Chen S.H.A., Makowski D. (2021). Heart Rate Variability in Psychology: A Review of HRV Indices and an Analysis Tutorial. Sensors.

[3] Waxenbaum J.A., Reddy V., Varacallo M. (2022). Anatomy, Autonomic Nervous System.

[4] Shaffer F., Ginsberg J.P. (2017). An Overview of Heart Rate Variability Metrics and Norms. Front. Public Health.

[5] Shaffer F., McCraty R., Zerr C.L. (2014). A Healthy Heart Is Not a Metronome: An Integrative Review of the Heart’s Anatomy and Heart Rate Variability. Front. Psychol..

[6] Baek H.J., Cho C.-H., Cho J., Woo J.-M. (2015). Reliability of Ultra-Short-Term Analysis as a Surrogate of Standard 5-Min Analysis of Heart Rate Variability. Telemed. E-Health.

[7] Task Force of the European Society of Cardiology the North American Society of Pacing Electrophysiology (1996). Heart Rate Variability: Standards of Measurement, Physiological Interpretation, and Clinical Use. Circulation.

[8] Laborde S., Mosley E., Mertgen A. (2018). Vagal Tank Theory: The Three Rs of Cardiac Vagal Control Functioning—Resting, Reactivity, and Recovery. Front. Neurosci..

[9] Shi H., Yang L., Zhao L., Su Z., Mao X., Zhang L., Liu C. (2017). Differences of Heart Rate Variability Between Happiness and Sadness Emotion States: A Pilot Study. J. Med. Biol. Eng..

[10] Geisler F.C.M., Vennewald N., Kubiak T., Weber H. (2010). The Impact of Heart Rate Variability on Subjective Well-Being Is Mediated by Emotion Regulation. Pers. Indiv. Differ..

[11] Bolger N., Laurenceau J.-P. (2013). Intensive Longitudinal Methods: An Introduction to Diary and Experience Sampling Research.

[12] Hamaker E.L., Wichers M. (2017). No Time Like the Present: Discovering the Hidden Dynamics in Intensive Longitudinal Data. Curr. Dir. Psychol. Sci..

[13] Fisher A.J., Medaglia J.D., Jeronimus B.F. (2018). Lack of Group-to-Individual Generalizability Is a Threat to Human Subjects Research. Proc. Natl. Acad. Sci. USA.

[14] Schwerdtfeger A.R., Gerteis A.K.S. (2014). The Manifold Effects of Positive Affect on Heart Rate Variability in Everyday Life: Distinguishing within-Person and between-Person Associations. Health Psychol..

[15] Kim J., Murata T., Foo J.C., Md Azmol Hossain B., Togo F. A Pilot Study of Temporal Associations Between Psychological Stress and Cardiovascular Response. Proceedings of the 2021 43rd Annual International Conference of the IEEE Engineering in Medicine & Biology Society (EMBC).

[16] van Halem S., van Roekel E., Kroencke L., Kuper N., Denissen J. (2020). Moments That Matter? On the Complexity of Using Triggers Based on Skin Conductance to Sample Arousing Events within an Experience Sampling Framework. Eur. J. Pers..

[17] Cohen S., Alper C.M., Doyle W.J., Treanor J.J., Turner R.B. (2006). Positive Emotional Style Predicts Resistance to Illness After Experimental Exposure to Rhinovirus or Influenza A Virus. Psychosom. Med..

[18] Csikszentmihalyi M. (1990). Flow: The Psychology of Optimal Experience.

[19] Fredrickson B.L. (2001). The Role of Positive Emotions in Positive Psychology. The Broaden-and-Build Theory of Positive Emotions. Am. Psychol..

[20] Body Position. https://docs.movisens.com/Algorithms/physical_activity/#body-position-bodyposition.

[21] Activity Class. https://docs.movisens.com/Algorithms/physical_activity/#activity-class-activityclass.

[22] Anastasopoulou P., Tansella M., Stumpp J., Shammas L., Hey S. Classification of Human Physical Activity and Energy Expenditure Estimation by Accelerometry and Barometry. Proceedings of the 2012 Annual International Conference of the IEEE Engineering in Medicine and Biology Society.

[23] Ainsworth B.E., Haskell W.L., Herrmann S.D., Meckes N., Bassett D.R., Tudor-Locke C., Greer J.L., Vezina J., Whitt-Glover M.C., Leon A.S. (2011). 2011 Compendium of Physical Activities: A Second Update of Codes and MET Values. Med. Sci. Sport. Exer..

[24] Energy expenditure. https://docs.movisens.com/Algorithms/energy_expenditure/#energy-expenditure.

[25] Welch P. (1967). The Use of Fast Fourier Transform for the Estimation of Power Spectra: A Method Based on Time Averaging over Short, Modified Periodograms. IEEE Trans. Audio Electroacoust..

[26] ECG, Heart Rate and Heart Rate Variability. https://docs.movisens.com/Algorithms/ecg_hr_hrv/#ecg-heart-rate-and-heart-rate-variability.

[27] Das-Friebel A., Lenneis A., Realo A., Sanborn A., Tang N.K.Y., Wolke D., Mühlenen A., Lemola S. (2020). Bedtime Social Media Use, Sleep, and Affective Wellbeing in Young Adults: An Experience Sampling Study. J. Child Psychol. Psychiatr..

[28] Watson D., Clark L.A., Tellegen A. (1988). Development and Validation of Brief Measures of Positive and Negative Affect: The PANAS Scales. J. Pers. Soc. Psychol..

[29] Russell J.A. (1980). A Circumplex Model of Affect. J. Pers. Soc. Psychol..

[30] R Core Team (2022). R: A Language and Environment for Statistical Computing.

[31] Benjamini Y., Hochberg Y. (1995). Controlling the False Discovery Rate: A Practical and Powerful Approach to Multiple Testing. J. Roy. Stat. Soc. B Met..

[32] Bates D., Mächler M., Bolker B., Walker S. (2015). Fitting Linear Mixed-Effects Models Using Lme4. J. Stat. Soft..

[33] Hottenrott L., Ketelhut S., Hottenrott K. (2019). Commentary: Vagal Tank Theory: The Three Rs of Cardiac Vagal Control Functioning—Resting, Reactivity, and Recovery. Front. Neurosci..

[34] Kanning M. (2013). Using Objective, Real-Time Measures to Investigate the Effect of Actual Physical Activity on Affective States in Everyday Life Differentiating the Contexts of Working and Leisure Time in a Sample with Students. Front. Psychol..

[35] Kanning M., Ebner-Priemer U., Brand R. (2012). Autonomous Regulation Mode Moderates the Effect of Actual Physical Activity on Affective States: An Ambulant Assessment Approach to the Role of Self-Determination. J. Sport Exerc. Psy..

[36] Li Y.-M., Hachenberger J., Lemola S. (2022). The Role of the Context of Physical Activity for Its Association with Affective Well-Being: An Experience Sampling Study in Young Adults. Int. J. Environ. Res. Public Health.

[37] Li Y.-M., Konstabel K., Mõttus R., Lemola S. (2022). Temporal Associations between Objectively Measured Physical Activity and Depressive Symptoms: An Experience Sampling Study. Front. Psychiatry.

[38] Wichers M., Lothmann C., Simons C.J.P., Nicolson N.A., Peeters F. (2012). The Dynamic Interplay between Negative and Positive Emotions in Daily Life Predicts Response to Treatment in Depression: A Momentary Assessment Study: Emotional Dynamics and Future Treatment Response. Brit. J. Clin. Psychol..

[39] Kok B.E., Fredrickson B.L. (2010). Upward Spirals of the Heart: Autonomic Flexibility, as Indexed by Vagal Tone, Reciprocally and Prospectively Predicts Positive Emotions and Social Connectedness. Biol. Psychol..

[40] Yerkes R.M., Dodson J.D. (1908). The Relation of Strength of Stimulus to Rapidity of Habit-Formation. J. Comp. Neurol. Psychol..

[41] Pauls C.A., Stemmler G. (2003). Repressive and Defensive Coping during Fear and Anger. Emotion.

[42] Quer G., Gouda P., Galarnyk M., Topol E.J., Steinhubl S.R. (2020). Inter- and Intraindividual Variability in Daily Resting Heart Rate and Its Associations with Age, Sex, Sleep, BMI, and Time of Year: Retrospective, Longitudinal Cohort Study of 92,457 Adults. PLoS ONE.

[43] Gilgen-Ammann R., Schweizer T., Wyss T. (2019). RR Interval Signal Quality of a Heart Rate Monitor and an ECG Holter at Rest and during Exercise. Eur. J. Appl. Physiol..

